# Application of Droplet-Array Sandwiching Technology to Click Reactions for High-Throughput Screening

**DOI:** 10.3390/mi16111270

**Published:** 2025-11-12

**Authors:** Yoshinori Miyata, Shoma Nishimura, Sora Kawakami, Yuriko Higuchi, Satoshi Konishi

**Affiliations:** 1 Graduate School of Science and Engineering, Ritsumeikan University, 1-1-1, Noji-higashi, Kusatsu 525-8577, Shiga, Japan; konishi@se.ritsumei.ac.jp; 2 Graduate School of Pharmaceutical Sciences, Kyoto University, 46-29, Yoshida-shimoadachi, Sakyo-ku, Kyoto 606-8501, Japan; nishimura.shoma.45z@st.kyoto-u.ac.jp (S.N.); kawakami.sora.75d@st.kyoto-u.ac.jp (S.K.); higuchi.yuriko.6v@kyoto-u.ac.jp (Y.H.); 3 College of Science and Engineering, Ritsumeikan University, 1-1-1, Noji-higashi, Kusatsu 525-8577, Shiga, Japan; 4 Ritsumeikan Global Innovation Research Organization, Ritsumeikan University, 1-1-1, Noji-higashi, Kusatsu 525-8577, Shiga, Japan

**Keywords:** high-throughput screening, click chemistry, microfluidics, droplet microarray, droplet-array sandwiching technology, wettability patterning

## Abstract

High-throughput screening (HTS) is an essential process in drug discovery, requiring platforms that ensure reagent economy, high efficiency, and resistance to cross-contamination. Click chemistry is well suited for HTS because of its biocompatibility, high selectivity, and quantitative fluorescent readout. We focus on droplet-array sandwiching technology (DAST), in which two droplet microarrays (DMAs) are vertically opposed to achieve solute transport and reagent mixing by controlled contact and separation. Herein, we integrate click chemistry with DAST and evaluate its feasibility as a HTS platform. In DAST, DMAs are formed on wettability-patterned (WP; hydrophilic/hydrophobic) substrates, preserving resistance to cross-contamination. First, we immobilized dibenzocyclooctyne (DBCO) on a WP substrate and verified the occurrence of DBCO–azide reaction using an azide-functional fluorescent dye. The fluorescence intensity increased with concentration and reached a plateau at higher concentrations, indicating saturation behavior in the DBCO–azide click reaction. Second, acoustic mixing with repeated droplet contact–separation was applied to generate concentration gradients on a single substrate while maintaining droplet independence. Third, we qualitatively reproduced the expected concentration dependence of manual handling by combining DAST-based gradient formation with click reaction fluorescence readout. These results reveal that DAST enables a reagent-efficient, cross-contamination-resistant, and low-instrument-dependent HTS foundation for click-chemistry-based assays.

## 1. Introduction

High-throughput screening (HTS) is a foundational technology through which reactions and efficacy evaluations for numerous compounds under diverse conditions are performed simultaneously to efficiently explore candidate compounds in drug discovery [1,2]. Consequently, HTS requires independent reaction chambers, rapid processing, and high-density arraying. Thus, experimental platforms that meet these requirements need to be developed [3,4].

The analytical chemistry characteristics required for HTS are biocompatibility under aqueous and mild conditions, high selectivity and rapid reaction rates, stable covalent bonding after washing, and quantitative readout via fluorescence. Click chemistry satisfies these requirements and is a promising model reaction for HTS [5,6]. It has been widely used as the base chemistry for surface immobilization and fluorescent microarraying; moreover, click chemistry-based HTS devices have been reported [7].

Microtiter plates are the most common format for the arrayed implementations of click reactions [8]. Although established as a standard HTS platform, these plates still consume considerable reagent volumes and typically depend on robotic systems, such as pipettors, dispensers, and plate handlers, for efficient operation [9]. Even for 1536-well plates, which reduce per-well volumes and screening costs, reliance on liquid-handling automation remains [10]. Against this backdrop, microfluidics has been advanced as a reagent-saving and high-efficiency platform for HTS [11].

Microfluidics manipulates nL–µL liquid volumes using structures and open surfaces on a scale ranging from several mm to a few hundred micrometers, enabling reduced reagent consumption, rapid reactions owing to short diffusion lengths, and parallel operations [12]. Among several microfluidic approaches, droplet microarray (DMA) is frequently reported for the arrayed implementations of click reactions [7,13].

DMA aligns microdroplets on an open surface and uses each droplet as an independent analytical chamber [14,15,16]. Dispensing techniques, such as microchannel cantilever spotting and inkjet printing, have enabled reagent-efficient and high-density array formation for DMA-based click reactions [17,18,19]. However, several issues remain, including possible cross-contamination owing to the coalescence of adjacent droplets on hydrophilic substrates [7]; the sensitivity of droplet shape and uniformity to dispensing conditions and surface roughness, which can limit reproducibility [20,21]; and reliance on specialized cantilevers and dispensing machinery [22].

Meanwhile, droplet-array sandwiching technology (DAST) employs DMA on wettability-patterned (WP) substrates—the combinations of hydrophilic and hydrophobic regions—to align and isolate droplets. In addition, it vertically opposes two arrays for the transportation, mixing, dispensing, and dilution of substances within droplets by controlled contact–separation [23,24]. The fusion of neighboring droplets is suppressed because droplets are confined by wettability barriers; a cross-contamination-resistant operation was reported with a barrier width of 175 µm [25,26]. Reagent consumption can be reduced by up to 3 × 10^4^-fold compared with 96-well methods, and very high-density arrays (~75 × 384-well plates per DMA substrate) can be constructed [25]. Moreover, DAST ensures the simultaneous manipulation, transportation, and dispension of multiple droplets without dedicated dispensers or large robots [23]. Previous studies reported rapid homogenization by acoustic waves, electric fields, and magnetic fields [27,28,29]; droplet volume control by electric fields [30]; and on-substrate gradient formation by simple stage control [27]. Biochemical applications, such as cell culture [31], intracellular calcium oscillations in droplets [32], and liquid crystal sensing [33], have also been reported. Recent studies have demonstrated droplet manipulation in viscoelastic environments, such as agarose and shear-thinning gels [34,35]. The DAST approach presented here may also be applicable to such systems in future studies. DAST operates in an open configuration rather than relying on oil encapsulation or channel confinement, which enables direct manipulation and reagent exchange on droplets [23]. This open accessibility allows for flexible experimental operation and makes DAST particularly suitable for assays that require manual intervention. These features position DAST as a platform that satisfies the HTS requirements of reagent economy, cross-contamination resistance, high-density arraying, and low instrument dependence.

**Figure 1:**
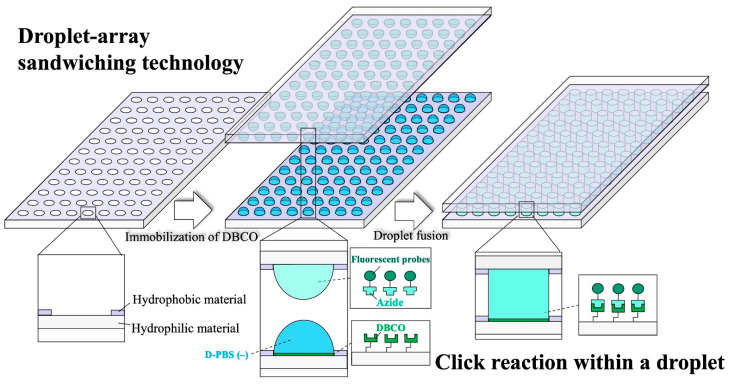
Conceptual diagram of a click reaction workflow in droplets using DAST. A WP substrate in which hydrophilic regions are surrounded by hydrophobic materials is fabricated to preserve droplet shape. DBCO is coated on WP as the click-reactive handle. D-PBS (−) is placed on the DBCO-functionalized WP. An upper DMA of azide-bearing fluorescent reagent is aligned and opposed. Contacting upper and lower droplets dispenses the fluorescent reagent and executes the in-droplet click reaction.

Herein, we apply DAST to click chemistry and demonstrate its compatibility with click reaction-based HTS (Figure 1). In particular, we incorporate a click-reactive handle (DBCO) into WP to verify reagent economical surface functionalization at the microliter scale. Next, we use DAST to generate a fluorescent reagent concentration gradient and perform simultaneous dispensing on a single substrate. Finally, we perform the strain-promoted azide–alkyne cycloaddition (SPAAC; DBCO–azide) reaction by batch dispensing on DBCO-functionalized WP to obtain a concentration–fluorescence intensity curve. DAST enables the evaluation of fluorescence intensity versus concentration on a single substrate. To the best of our knowledge, this is the first study to incorporate click reaction handles into DMA substrates using WP and conduct the reaction thereon. Among click reactions, SPAAC, which occurs in H_2_O without catalysts [36], was adopted as a model reaction in this study.

## 2. Materials and Methods

### 2.1. Materials and Reagents

Slide glass (Matsunami Glass Ind., Ltd., Osaka, Japan); Cytop^TM^ (AGC Inc., Tokyo, Japan); OFPR-800LB (Tokyo Ohka Kogyo Co., Ltd., Kanagawa, Japan); NH_3_ (28%), H_2_O_2_ (35%), H_2_SO_4_ (95%), EtOH (99.5%), and NaHCO_3_ (Nacalai Tesque, Inc., Kyoto, Japan); DMSO (FUJIFILM Wako Pure Chemical Corp., Osaka, Japan); D-PBS (–) (Shimadzu Diagnostics Corp., Tokyo, Japan); (3-mercaptopropyl)trimethoxysilane (MPTMS; Tokyo Chemical Industry Co., Ltd., Tokyo, Japan); MAL-PEG-NH_2_ (MW 2000) and mPEG-MAL (MW 2000) (Creative PEGWorks, Chapel Hill, NC, USA); DBCO-PEG5-NHS ester and Azide-Fluor 488 (Click Chemistry Tools, Scottsdale, AZ, USA); 10 cm dishes (Corning Inc., Corning, NY, USA); storage containers (SANPLATEC Corp., Osaka, Japan); and a dust blower (SANWA SUPPLY Inc., Okayama, Japan) were used.

### 2.2. Fabrication of WP Substrates

**Figure 2:**
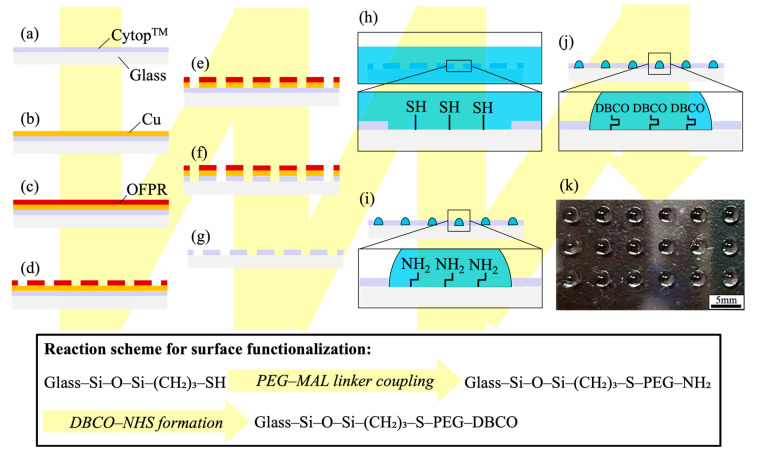
Schematic of WP substrate fabrication and immobilization of click-reactive groups: (**a**–**g**) WP fabrication, (**h**–**j**) DBCO immobilization on WP, and (**k**) photograph of D-PBS (−) droplets formed on the finished substrate. A simplified reaction scheme for surface functionalization corresponding to panels (**h**–**j**) is shown at the bottom.

Figure 2 shows the fabrication of WP substrates and the subsequent immobilization of click-reactive groups. After spin-coating Cytop^TM^ on glass (Figure 2a), Cu (100 nm) was thermally evaporated (Figure 2b). OFPR-800LB was spin-coated (Figure 2c), photolithography was performed (Figure 2d), and Cu was selectively removed via wet etching (Figure 2e). Exposed Cytop^TM^ was removed by O_2_ plasma (Figure 2f), and the residual photoresist and Cu were stripped (Figure 2g). Circular glass surfaces surrounded by Cytop^TM^ served as hydrophilic regions, and an 18-pattern array (3 × 6) was designed. The upper and lower substrates had WP diameters of 2.80 and 2.48 mm, respectively, to avoid volume change during contact–separation, a design based on previous research showing that droplet volume can be controlled by radius differences between the upper and lower droplets [37]. To maintain droplet height during DAST operation, the WP substrate (2.48 mm in diameter) was designed so that a 4 µL droplet forms a hemispherical shape with a contact angle of approximately 90° [38].

### 2.3. Cleaning and Silanization

WP substrates were baked at 160 °C for 1 h to improve hydrophobicity, ultrasonically cleaned in NH_3_:H_2_O_2_:H_2_O = 50:50:250 (*v*/*v*, 350 mL) for 10 min, and ultrasonically cleaned twice with EtOH (3 min each). They were then immersed in H_2_O_2_:H_2_SO_4_ = 80:240 (*v*/*v*, 320 mL) for 120 min, rinsed with H_2_O, and ultrasonically washed in H_2_O and EtOH (3 min × 2 each). Silanization was performed by immersing substrates in 1% (*v*/*v*) MPTMS in EtOH containing 4% (*v*/*v*) H_2_O for 24 h at room temperature (approximately 25 °C) in storage containers, which were sealed with Parafilm and protected from light (Figure 2h). After EtOH rinsing, the substrates were baked at 110 °C for 1 h.

### 2.4. PEG Modification

Solutions (2 mg/mL) of MAL-PEG-NH_2_:mPEG-MAL = 30:70 (*w*/*w*) or mPEG-MAL alone were prepared in D-PBS (−) via 15 min ultrasonication and dispensed on 3 × 6 WP substrates (4 µL per pattern). Only mPEG-MAL was used as a control in column 1 (3 patterns), whereas a mixture of MAL-PEG-NH_2_ and mPEG-MAL was used in columns 2–6 (15 patterns). The substrates were incubated in 10 cm dishes for 24 h at room temperature (approximately 25 °C) under light-shielding and evaporation-suppressing conditions (Figure 2i). After incubation, droplets were aspirated and two washing cycles were performed with D-PBS (−) (4 µL per pattern, dispense→aspirate).

### 2.5. DBCO Immobilization

DBCO-PEG5-NHS was dissolved in DMSO and then added to 50 mM NaHCO_3_ (pH 8.3) to prepare a 3.6 mM solution (10 s vortex). The resulting solution (4 µL) was dispensed onto 30:70 MAL-PEG-NH_2_:mPEG-MAL-modified patterns, whereas D-PBS (−) (4 µL) was dispensed onto mPEG-MAL-modified patterns (Figure 2j). After a 1 h reaction at room temperature (approximately 25 °C) under light-shielded and evaporation-suppressed conditions, droplets were aspirated and washed twice with D-PBS (−) (4 µL per pattern, dispense→aspirate). Figure 2k shows 4-µL D-PBS (−) droplets formed on the DBCO-functionalized WP substrates; the droplets were aligned on the 3 × 6 WP substrates without mixing between neighbors.

### 2.6. DBCO–Azide Click Reaction

#### 2.6.1. Manual Method

To verify DBCO immobilization on WP, Azide-Fluor 488 was dispensed by manual pipetting, and the presence/absence of DBCO–azide reaction was evaluated. Azide-Fluor 488 was prepared as a 5 mM DMSO stock, and diluted with D-PBS (–) to final concentrations of 10/20/40/80 µM. Column 1 received 80 µM mPEG-MAL (4 µL), column 2 received D-PBS (–) (4 µL), and columns 3–6 received 10/20/40/80 µM Azide-Fluor 488 solutions (4 µL each). Reactions proceeded for 15 min at room temperature (approximately 25 °C) under light-shielding and evaporation-suppressing conditions.

#### 2.6.2. DAST Method (Gradient Generation and Batch Dispensing)

We extended our previously reported approach—uniform mixing by acoustic waves during repeated contact–separation [27]—to a droplet-array system to generate concentration gradients. A speaker (SC 4.6 FL-8 OHM, Visaton GmbH & Co. KG, Haan, Germany) provided agitation, and an electric X–Y stage (KXL06050-N1-CA, Suruga Seiki Co., Ltd., Shizuoka City, Japan) and Z stage (KHE06008-C, Suruga Seiki Co., Ltd., Shizuoka City, Japan) were positioned at the upper substrate. An AC voltage from a waveform generator (33210A, Keysight Technologies, Santa Rosa, CA, USA) was applied to the speaker at 5.65 Vpp and 200 Hz. During each fusion step in DAST, agitation was applied for 30 s to homogenize the upper and lower droplets. The contact–separation speed of the droplets was controlled at approximately 2.5 mm/s using a motorized stage.

Azide-Fluor 488 was prepared as a 5 mM DMSO stock and diluted to 320 µM with D-PBS (−). On the lower substrate, column 1 (3 patterns per row) received 4 µL Azide-Fluor 488; on the upper substrate, columns 2–6 received D-PBS (−) (4 µL). After 30 s of standard agitation, the two substrates were fused column-wise and separated in the order of 6→5→4→3 to form a 1:1 serial dilution. This produced 0/20/40/80/160 µM (4 µL each) on columns 2–6 of the upper substrate; column 2 served as D-PBS (−), and column 1 remained untreated. Slight evaporation in open-surface droplet systems can affect solute distribution and concentration gradients [39,40]. However, the droplet volume used in this study was relatively large (4 µL), and the total operation time was short (<3 min), so the evaporation does not cause significant influence in this study.

Fresh DBCO-functionalized lower substrates (column 1 = mPEG-MAL and columns 2–6 = 30:70 MAL-PEG-NH_2_:mPEG-MAL) were prepared, and all patterns except column 1 received D-PBS (–) (4 µL). Column 1 received 80 µM Azide-Fluor 488 (4 µL), which was prepared separately. Then, the concentration-adjusted upper substrate and DBCO-functionalized lower substrate were fused column-wise (1:1), yielding 0/10/20/40/80 µM Azide-Fluor 488 solutions (4 µL each) on the DBCO-functionalized substrate (column 2 = D-PBS (–)). Substrates were reacted for 15 min at room temperature (approximately 25 °C) under light-shielding and evaporation-suppressing conditions. To confirm concentration gradients, absorbance–concentration correlations were measured using a micro-UV–Vis spectrophotometer (NanoDrop One, Thermo Fisher Scientific Inc., Waltham, MA, USA).

#### 2.6.3. Post-Reaction Washing

For both manual and DAST methods, the reaction solutions were aspirated, followed by two washing cycles with D-PBS (–) (4 µL, dispense→aspirate) and one rinse each with EtOH and distilled water. The substrates were then dried using a dust blower.

### 2.7. Fluorescence Imaging and Analysis

#### 2.7.1. Whole-Substrate Imaging (LuminoGraph I)

Whole-substrate fluorescence images were acquired using a LuminoGraph I (WSE-6100H-ACP, ATTO Corporation, Tokyo, Japan) with transmitted cyan illumination and a YA-3 filter, normal (1 × 1) mode, 2 s exposure, and 16-bit (ImageSaver 6). Auto-exposure was disabled. Images were converted to 8-bit in ImageJ 1.54g. A uniform background of 18 gray levels (8-bit scale, 0–255) was subtracted from all pixels using the Subtract function in ImageJ to correct for a nearly uniform baseline signal across the substrate. TIFF files were then saved. Circular ROIs (18 px diameter) were placed within each pattern, and the mean gray values were calculated.

#### 2.7.2. Per-Pattern Microscopy (Inverted Microscope)

Fluorescence images were acquired using an inverted microscope (IX73, Olympus Corporation, Tokyo, Japan) with a CMOS camera (filter U-FBNA: BP470–495/BA510–550/DM505; light source U-HGLGPS at 25%), objective UPlanFL N 4×/0.13, 600 ms exposure, and 9.82 gain (AdvanView ×64 v4.12). Bright-field images were acquired using the same objective at 20 ms exposure and 1.00 gain.

## 3. Results and Discussion

### 3.1. Evaluation of DBCO Immobilization on WP

**Figure 3:**
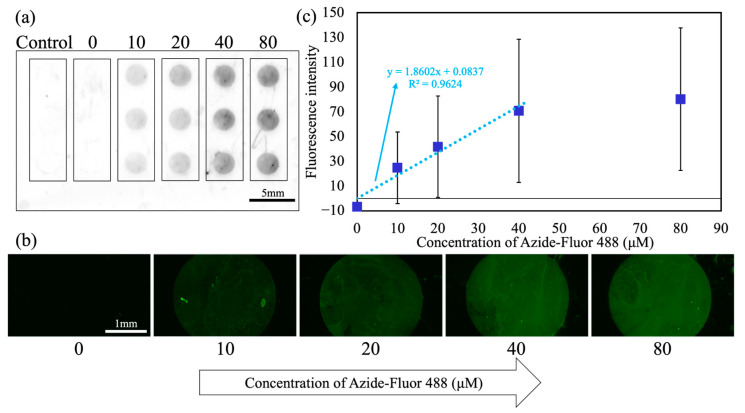
Evaluation of DBCO–azide click reactions on WP using the manual method: (**a**) representative whole-substrate fluorescence image, (**b**) fluorescence micrographs for the first row of (**a**), and (**c**) quantitative fluorescence analysis; N = 15 patterns per concentration. Blue squares indicate experimental data points, and the light blue line represents the linear fit.

Figure 3 shows the results of the DBCO–azide click reactions using the manual method. Azide-Fluor 488 solutions were manually dispensed and their reaction with immobilized DBCO was evaluated to confirm DBCO immobilization on WP. A representative whole-substrate fluorescence image is shown in Figure 3a. No fluorescence was observed in the mPEG-MAL control (column 1) or at 0 µM (column 2 = D-PBS (–)). Meanwhile, clear fluorescence was detected at 10–80 µM (columns 3–6), indicating successful DBCO immobilization and reaction on the substrate. The droplets retained their circular shapes after washing, confirming the effective retention and isolation by WP. Figure 3b shows the fluorescence micrographs for the first row in Figure 3a. The aforementioned trend was observed: negligible fluorescence at 0 µM, followed by concentration-dependent increases at 10–40 µM, with an apparent saturation beyond ~40 µM. Quantitative analysis (Figure 3c) yielded a linear fit in the lower concentration range of 10–40 µM (y = 1.8602x + 0.0837, R^2^ = 0.9624). For each concentration condition, fluorescence intensities were obtained from 15 droplet sites (3 rows per substrate × 5 independently prepared DBCO-functionalized substrates). The mean fluorescence values and standard deviations were calculated from these 15 data points, and each plotted value represents the mean fluorescence intensity averaged over a circular ROI placed within each droplet.

In our results, the concentration–fluorescence intensity curve increased steeply at lower concentrations and plateaued at higher concentrations, consistent with the saturation-type Langmuir responses driven by the finite number of surface-binding sites [41,42]. At concentrations above 40 µM, the fluorescence intensity showed little further increase. This minimal increase may be attributed to the saturation of binding. Notably, reaction kinetics were not observed here per se but at the bound amount at (near) equilibrium, consistent with the characteristic concentration–response curve of the Langmuir model. Fluorescence intensity–concentration curves similarly behaved in click reaction arrays [7,43]. Furthermore, in the coating process, the required solution volumes for PEG modification and DBCO immobilization were limited to 4 µL per pattern. Compared with previous research using a 24-well plate experiment (500 µL per well) [36], this limitation enabled a reagent saving of 496 µL per reaction, corresponding to a 99.2% reduction in reagent consumption.

### 3.2. Concentration Gradient Generation by DAST

**Figure 4:**
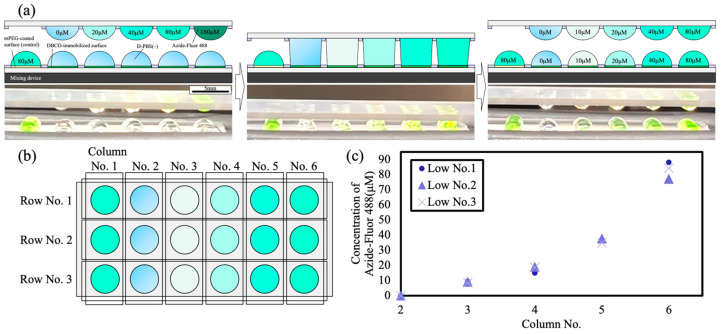
Concentration gradient generation on droplet arrays using DAST: (**a**) concept and photographs showing half of the concentration upon fusion of an Azide-Fluor 488 droplet with a D-PBS (−) droplet, (**b**) post-dispense layout on the DBCO-functionalized WP, and (**c**) analysis of fluorescent reagent concentrations for each row.

Figure 4 shows the results of gradient generation using DAST. We extended an acoustic-mixing-assisted contact–separation protocol [27] to a droplet-array system, enabling the control of solute concentrations within droplets across multiple rows. Using the procedure in Section 2.6.2, a gradient of Azide-Fluor 488 was formed in the upper droplets, while directly opposite on the DBCO-functionalized WP, D-PBS (–) droplets were formed beneath the upper droplets. As shown in Figure 4a, contacting azide-containing upper droplets with D-PBS (−) lower droplets, mixing acoustically, and separating them stepwise generated a controlled gradient on the DBCO-functionalized WP. After separation, the lower droplets exhibited fluorescence with the color intensity varying with concentration, which was visible.

As shown in Figure 4b,c, droplets across multiple rows and columns matched the target concentrations, confirming accurate gradient generation by DAST. This approach produces a matrix of droplets with different concentrations on a single substrate and enables the simultaneous dispensing of different reagent concentrations, offering a high-efficiency alternative to conventional manual pipetting. Each acoustic-assisted contact–separation cycle required approximately 30 s, which is much shorter than the 15 min reaction time of the SPAAC process. Therefore, the reaction progress during gradient formation was considered to have a negligible effect on dilution accuracy.

### 3.3. Click Reactions Executed by DAST

**Figure 5:**
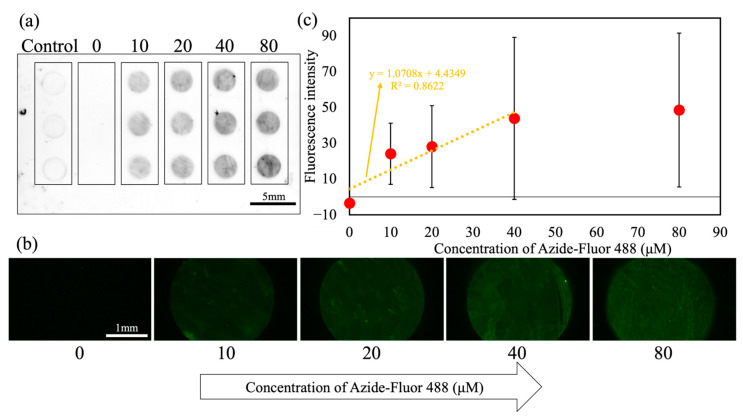
Evaluation of DBCO–azide click reactions on WP executed via DAST: (**a**) representative whole-substrate fluorescence image, (**b**) fluorescence micrographs for the first row of (**a**), and (**c**) quantitative fluorescence analysis; N = 15 patterns per concentration. Red circles indicate experimental data points, and the orange line represents the fitted curve.

Figure 5 shows the click reaction results using DAST. In the whole-substrate image (Figure 5a), no fluorescence was observed at 0 µM (column 2), while clear fluorescence was detected at 10–80 µM (columns 3–6), confirming the DBCO–azide click reactions at the immobilized sites. The droplets retained their circular shapes after washing, indicating that wettability barriers suppressed fusion with neighbors. Weak peripheral fluorescence was observed in the mPEG-MAL column (column 1), attributable to the nonspecific adsorption of dye on the hydrophobic Cytop^TM^ surface rather than a reaction at binding sites.

Fluorescence micrographs (Figure 5b) demonstrated increasing intensities in the range of 10–40 µM, followed by saturation in the range of 40–80 µM. Quantitative analysis (Figure 5c) yielded a linear fit in the 10–40-µM range (y = 1.0708x + 4.4349, R^2^ = 0.8622). Fluorescence intensities for each concentration condition were obtained from 15 droplet sites (3 rows per substrate × 5 independently prepared DBCO-functionalized substrates). The mean fluorescence values and standard deviations shown in Figure 5c were calculated from these 15 data points, and each plotted value represents the mean fluorescence intensity averaged over a circular ROI placed within each droplet. A certain degree of variation in fluorescence intensity was observed in both manual and DAST operations; however, similar fluctuations were also found in the manually operated assays, indicating that the variation originates mainly from the intrinsic heterogeneity of the click reaction and the DBCO immobilization processes rather than from DAST manipulation.

The fluorescence responses obtained using DAST exhibited concentration-dependent trends consistent with Langmuir-type behavior and qualitatively matched those observed in the manual method. Thus, DAST allows for the evaluation of concentration-dependent fluorescence in click reactions and is compatible with click-chemistry-based HTS.

In this study, a 3 × 6 droplet array was used as a minimal configuration to demonstrate the operational feasibility and chemical compatibility of DAST. However, the system can be easily scaled up by modifying the wettability-patterned design and substrate size. Popova et al. [25] reported droplet microarrays based on superhydrophobic–superhydrophilic patterning that achieved droplet densities equivalent to approximately 75 × 384-well plates on a single substrate. Because DAST is based on a similar patterning concept, the 18-droplet array presented here serves as a proof-of-concept configuration, with the potential to be extended toward high-density and high-throughput screening applications in future studies.

In conventional dispensing systems, increasing the number of patterns prolongs the overall dispensing time, potentially delaying the initiation of the reaction. In contrast, DAST performs simultaneous contact and separation of all droplet pairs by aligning two wettability-patterned substrates. Thus, even when the array size increases, the total operation time before the reaction remains nearly constant. In this study, the click reaction was conducted as a single simultaneous contact–separation operation of the preadjusted droplet arrays, eliminating time-dependent constraints such as overreaction during dispensing. This batchwise operation represents a key practical advantage of DAST over conventional sequential dispensing methods.

Furthermore, DAST enables the evaluation of concentration-dependent fluorescence responses in click reactions and demonstrates its applicability to biochemical assays. Because DAST operates as an open microarray platform, it allows for versatile operations such as direct access to droplets and the integration of surface electrodes. Recent studies have reported that open-droplet-array systems can be extended beyond fluorescence detection to other analytical modes, including colorimetric, electrochemical, and mass-spectrometric analyses [44]. These findings suggest that DAST has strong potential to evolve into a versatile platform technology applicable to a wide range of analytical and biochemical assay systems.

## 4. Conclusions

We demonstrated a reagent-efficient, cross-contamination-resistant, and low-instrument-dependent HTS foundation by applying DAST to click chemistry. First, DBCO was immobilized on WP substrates and validated via the DBCO–azide reaction using Azide-Fluor 488, yielding fluorescence that increased at low concentrations and approached a plateau at higher concentrations, indicating saturation behavior consistent with previously reported click reaction array trends. Second, we extended an acoustic-mixing-assisted concentration control method to droplet arrays and verified DAST-based gradient generation, enabling accurate control of multiple concentration conditions on a single substrate without relying on micropipetting or dispensing robots. Third, DAST-executed SPAAC reactions reproduced the qualitative concentration dependence observed in manual handling. Collectively, these results reveal that DAST is compatible with click-chemistry-based HTS and enables the evaluation of concentration-dependent fluorescence in click reactions. In the future, we will focus on extending DAST-based click reactions to cell-based HTS and more complex chemical screening schemes. As a future perspective, automating the contact–separation operations in DAST is expected to enable more precise and reproducible droplet manipulation. Developing such an automated control will be a key step toward establishing DAST as a scalable and fully integrated high-throughput screening platform. We expect these advances to contribute to the further development of click-chemistry-based HTS.

## Data Availability

All data generated or analyzed during this study are included in this published article.
